# Temporal sensitivity for achromatic and chromatic flicker across the visual cortex

**DOI:** 10.1101/2023.07.24.550403

**Published:** 2023-07-26

**Authors:** Carlyn Patterson Gentile, Manuel Spitschan, Huseyin O. Taskin, Andrew S. Bock, Geoffrey K. Aguirre

**Affiliations:** 1 University of Pennsylvania; 2 Children’s Hospital of Philadelphia; 3 Translational Sensory & Circadian Neuroscience, Max Planck Institute for Biological Cybernetics, Tübingen, Germany; 4 Chronobiology & Health, TUM Department of Sport and Health Sciences (TUM SG), Technical University of Munich, Munich, Germany

## Abstract

The classes of retinal ganglion cells (RGCs) receive different combinations of L, M, and S cone inputs and give rise to one achromatic and two chromatic post-receptoral channels. Beyond the retina, RGC outputs are subject to filtering and normalization along the geniculo-striate pathway, ultimately producing the properties of human vision. The goal of the current study was to determine temporal sensitivity across the three post-receptoral channels in subcortical and cortical regions involved in vision, to better characterize post-retinal temporal processing. We measured functional magnetic resonance imaging (MRI) responses at 7 Tesla from participants viewing a high-contrast, flickering, spatially-uniform wide (~140°) field. Stimulus flicker frequency varied logarithmically between 2 and 64 Hz and targeted the L+M+S, L–M, and S–[L+M] cone combinations. These measurements were used to create temporal sensitivity functions (TSFs) of primary visual cortex (V1) across eccentricity, and spatially averaged responses from lateral geniculate nucleus (LGN), V2/V3, hV4, and MT. Functional MRI responses reflected known properties of the visual system, including higher peak temporal sensitivity to achromatic vs. chromatic stimuli, and low-pass filtering between the LGN and V1. V1 had the slowest peak temporal sensitivity across cortical regions, which increased at higher levels of the visual cortical hierarchy. Unexpectedly, peak temporal sensitivity decreased at greater eccentricities in area V1, especially for achromatic stimuli. Comparison of measured cortical responses to a model of integrated retinal output to our stimuli demonstrates that extensive filtering and amplification is applied to post-retinal signals.

## Introduction

Cone and retinal ganglion cell (RGC) density declines exponentially from the fovea to the periphery. There is a corresponding and well-studied change in the spatial frequency sensitivity (acuity) of human perception that reflects this change with eccentricity (Thibos et al., 1996). Although less numerous, cell populations in the peripheral retina are found to have faster kinetics (Seiple and Holopigian, 1996; Solomon, 2005), raising the possibility that visual perception has a corresponding change in temporal frequency sensitivity with eccentricity.

**Figure 1.**
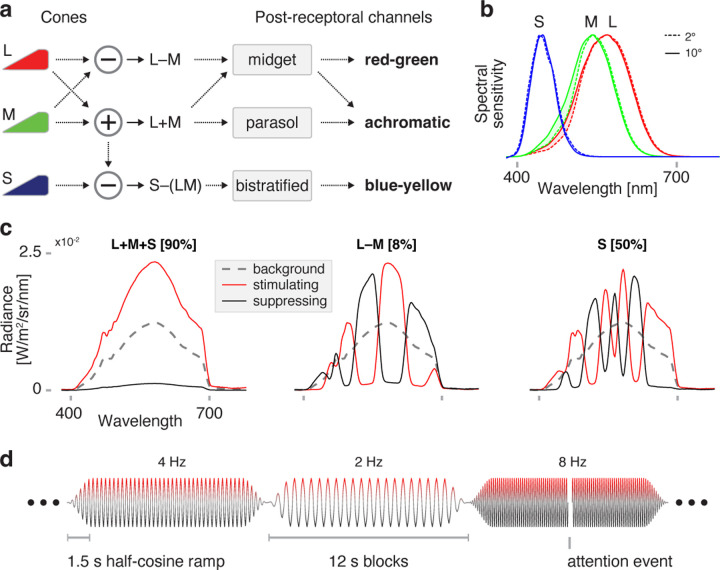
Targeting the post-receptoral channels with spectral flicker (a) L-, M-, and S-cones interact at the level of the retina to form the achromatic (L+M), chromatic red-green (L–M), and chromatic blue-yellow (S–[L+M]) post-receptoral channels. Midget RGCs contribute substantially to both the achromatic and red-green channels. (b) The spectral sensitivities of the cones vary slightly between central (2 degrees, dotted line) and more peripheral (10 degrees, solid line) eccentricity locations, largely due to the filtering effects of macular pigment. (c) Spectral modulations were generated to target combinations of cone classes while silencing others, corresponding to the three cardinal axes of DKL space. Stimuli modulated between a suppressing (black) and stimulating (red) spectrum, around a common background (dotted gray). Different modulations produced (*left*) equal nominal stimulation of the L, M, and S cones, thus producing isolated stimulation of the L+M channel, (*center*) differential stimulation of the L and M cones, while silencing S, and (*right*) isolated stimulation of the S cones, producing targeted stimulation of the S-[L+M] channel. Nominal Michelson contrast upon the targeted post-receptoral channels is given in brackets above each plot. Spectral targeting accounted for the 2° and 10° variants of spectral sensitivity for the cone classes, in an attempt to equate stimulus contrast across the visual field. (d) Temporal flicker was presented at logarithmically spaced frequencies between 2 Hz and 64 Hz (as well as 0 Hz) in each of many 12 second trials. The modulation was subjected to a half-cosine ramp at onset and offset. The ordering of the stimulus frequencies was specified by a pseudo-random, counterbalanced sequence. During intermittent attention events the entire stimulus field dimmed for 250 msecs, in response to which the participant was asked to make a bilateral button press. Different fMRI acquisitions presented modulations that targeted a different post-receptoral channel.

Signals from the cones are combined into three post-receptoral channels (Figure 1a): one achromatic or luminance (L+M) channel, and two chromatic channels, red-green (L-M), and blue-yellow (S–[L+M]). The eccentricity dependence of temporal sensitivity has been most studied for achromatic stimuli, and generally shows an improvement in threshold contrast detection for higher frequency flicker in peripheral as compared to central vision (Snowden and Hess, 1992). In foveal vision, chromatic channels favor slower frequencies as compared to the achromatic channel (Swanson et al., 1987). Relatively little is known regarding how eccentricity variation influences temporal sensitivity across the chromatic post-receptoral channels, although one set of measurements made in the LM plane out to 14° found little variation in temporal response (Gelfand and Horwitz, 2018).

The properties of threshold detection as measured with psychophysics may differ in meaningful ways from the perceptual experience of supra-threshold stimuli (i.e., natural vision). Neural responses to supra-threshold stimuli might be expected to reflect the properties of visual perception. Electrophysiological studies in non-human primates demonstrate filtering of temporal information at successive stages of the visual system; the largest decline in temporal sensitivity occurs between the cones and RGCs, with additional filtering of high-temporal frequency information between the lateral geniculate nucleus (LGN) and cortex (Hawken et al., 1996; Horwitz, 2020, 2021). Consequently, we may expect that the temporal sensitivity of human visual cortex—and presumably human supra-threshold perception—reflects a substantial modification of the initial retinal signal.

Here we report measurements of the regional and eccentricity dependence of the temporal sensitivity of human visual cortex. We studied two participants using a 7 Tesla scanner while they viewed high-contrast, wide-field stimuli that targeted each of the three post-receptoral channels, modulating across a range of flicker frequencies. Our results provide temporal sensitivity functions across multiple brain regions representing stages of the visual pathway from lateral geniculate nucleus (LGN) to extra-striate cortex. More detailed measurements across eccentricity were also obtained for the primary visual cortex (area V1). Our findings confirm several temporal filtering properties of the visual system that have been previously reported, as well as normalization of signals in area V1 across post-receptoral channel and eccentricity relative to the retina and LGN. Unexpectedly, we find a slowing of temporal sensitivity to achromatic flicker at far eccentricities.

## Materials and Methods

### Participants

The two participants are authors of this report (P1, age 46; P2 age 32, both male). Both participants had normal or corrected to normal acuity and normal color vision. This study was approved by the University of Pennsylvania Institutional Review Board in accordance with the Declaration of Helsinki, and all participants provided written consent.

### Custom contact lens

The participants underwent clinical refraction and measurement of keratometry. These measures were then used to design custom contact lenses for each participant that provided either +36 or +32 diopters of magnification, with the latter selected to complement the −4 diopter myopia for that participant. The contact lenses were Alden Optical, HP54 spherical soft lenses, constructed from 54% Hioxifilcon D (sourced from Benz Research and Development). The lens was 0.59 mm at its thickest point. During scanning, the participant wore a contact lens in the right eye. Prior to each scanning session, the participant underwent pharmacologic dilation of their right eye; the left eye was patched.

### Spectral modulations

Stimuli were generated using a digital light synthesis engine (OneLight Spectra). This device produces desired spectra through the use of a digital micro-mirror device chip, and was configured to mix 56 independent primaries with a FWHM of ~16 nm at a refresh rate of 256 Hz. Spectral modulations were designed to target specific photoreceptor classes alone or in combination, using the silent substitution approach (Estévez and Spekreijse, 1982). The design of these stimuli has been described in detail previously (Spitschan et al., 2015; Spitschan and Woelders, 2018; Barnett et al., 2021). Briefly, a set of three modulations were created, which targeted L–M, S, and LMS cone combinations; the last of these was a “light flux” modulation, in which the entire stimulus spectrum was scaled. All three stimulus types were modulations around the half-on background of the device.

The chromatic L–M and S modulations were designed to minimize differences in contrast between the fovea and the periphery. This was achieved by treating the cone fundamentals at 2 and 10 degrees (CIE, 2006) as different classes of photoreceptors, and tailoring modulations to produce equivalent contrast on these two classes (Barnett et al., 2021). The cone fundamentals were adjusted for age-related differences in lens density (CIE, 2006), and the transmittance spectrum of the contact lens material was measured and incorporated into the model of pre-receptoral filtering.

Spectroradiometric measurements of the stimuli were made before and after each experimental session (PhotoResearch PR-670). The nominal maximum contrast of the modulation was approximately 90% for LMS, 50% for S, and 8% for L–M. Supplementary Figure 1 and Supplementary Table 1 provide the predicted contrast of the stimuli as a function of eccentricity and in the presence of imprecision in device control. The chromaticity of the background was x = 0.4025, y = 0.4486, luminance Y = ~400 cd/m^2^; chromaticity and luminance computed with respect to the XYZ 10° physiologically relevant color matching functions (https://cvrl.org). Assuming a 7mm diameter of the pharmacologically dilated eye, the stimulus background was ~4200 Trolands.

The stimuli were conveyed via a fiber-optic cable to a custom-made, MR-compatible eye piece that was positioned over the right eye of the participant. After passing through ultra-violet and heat-absorbing glass, light was back-projected on a 35 mm diameter, opal diffusing glass. The stimulus surface was positioned ~10 mm from the eye. This geometry provided a uniform stimulus field of ~60° radial eccentricity. The +36 diopter contact lens produced approximately x1.18 spectacle magnification. The resulting stimulus field was therefore close to 70° radial eccentricity.

### Scanning environment and MR imaging of the brain

An MR imaging session was conducted for each participant on each of two consecutive days. During the scanning session, all sources of light within the scanner room were extinguished and covered. Room lights were on when participants were positioned within the scanner, but these were turned off at the start of data acquisition.

All MRI images were obtained with a 32-channel head coil on a 7T Siemens scanner. The anatomical T1w images were acquired with voxel size=0.8 mm iso, resolution = 480×640×176, TR = 2800ms, TE=4.28ms, TI=1500ms, and flip angle=7°. For the BOLD images, a multiband epfid2d1_130 sequence was used with multiband acceleration factor=5, voxel size=1 mm iso, resolution=130×130×75, TR=1000ms, TE=26.8ms, flip angle=45°, and average=1.

### Experimental design

Each fMRI acquisition was 336 seconds in duration and presented modulations that targeted one particular post-receptoral channel (LMS, L–M, or S). The acquisition was composed of 28, 12 second trials. Each trial presented a spectral modulation that flickered at one of 7, log-spaced frequencies (0, 2, 4, 8, 16, 32, 64 Hz). A pseudo-random, counterbalanced ordering of the frequencies was defined (50 elements). This sequence was divided into two-subsequences (“A” and “B”), each padded with three instances of the 0 Hz trial at the end. A set of 6 acquisitions composed a scanning “block”, and consisted of the ordered acquisitions: LMS_A, L–M_A, S_A, S_B, L–M_B, LMS_B. A total of 6 blocks were collected for each participant across the two sessions. The stimulus field was set to the half-on background before and between each acquisition.

The sinusoidal spectral modulation presented in each block was windowed by a 1.5 second, half-cosine ramp at trial onset and offset. An intermittent attention event occurred with a 33% probability during each trial. The attention event could occur at any point during the trial, except constrained not to take place during first or last 2 seconds of the trial. The attention event was a 250 msec darkening of the stimulus field. The participant was asked to make a bilateral button press on a fiber-optic pad in response to each attention event. Performance on this attention task was uniformly high for the two participants.

### Pre-processing of fMRI data

Three-dimensional surface reconstruction of the anatomical images for both participants was performed using Freesurfer (Dale et al., 1999). For the preprocessing of the BOLD images, we used the *fmriprep* pipeline with custom options (Esteban et al., 2019). The pipeline included bias field correction (Tustison et al., 2010), brain extraction and normalization (Avants et al., 2008), tissue segmentation (Zhang et al., 2001), slice timing correction (Cox, 1996), and motion correction (Jenkinson et al., 2002). As no B0 field maps were collected, we performed susceptibility correction with symmetric group-wise diffeomorphic normalization (*SyN*). This method aims to correct distortion by inverting the intensity of BOLD images (Huntenburg, 2014; Wang et al., 2017) and registering these inverted images to subject T1w image using an average fieldmap template as the constraint (Treiber et al., 2016). Noise regressors were obtained with AROMA (Pruim et al., 2015). The set of regressors was inspected and any component with a spatial extent that was largely confined to the occipital cortex was excluded. The effect of the remaining set of regressors was removed from the data using the “non-aggressive” denoising strategy. Finally, we mapped the preprocessed volumetric BOLD data to HCP CIFTI FSLR_32k template space with *ciftify* which uses the algorithms and templates from the HCP minimal processing pipeline (Glasser et al., 2013) to map volumetric timeseries data to CIFTI surfaces. Because we did not expect, and did not observe, response differences between the hemispheres, we averaged the data from the left and right hemisphere into a single “pseudo” hemisphere.

### Modeling of fMRI time-series data

The time-series data in each voxel was fit with a non-linear model that simultaneously estimated the amplitudes of response to the stimuli and the shape of the hemodynamic response function. First, the time-series data in each voxel in each acquisition were converted to percentage change units. The data from all acquisitions across both sessions were then concatenated, for a total of 12,096 time points. The amplitude of response to each stimulus frequency was modeled as a 12 second step function. Attention events were modeled as delta functions. All trials of a given stimulus frequency in a single acquisition were modeled with a single covariate, but different covariates modeled the response in each acquisition (for a total of 8×36 = 288 covariates). The covariates were subject to convolution by a model of the hemodynamic response function, which itself was under the control of three parameters that specify the weighted combination of an orthogonal basis (Woolrich et al., 2004). The form of the HRF was held in common across all acquisitions for a given voxel for a given participant. Within a non-linear model fitting routine the shape of the HRF and the amplitudes of the covariates were adjusted to minimize the L2 norm of the difference between the observed and modeled fMRI time series, after subjecting the model and data to a 0.0387 Hz high-pass filter.

This analysis yielded estimates of the amplitude of neural response to each stimulus frequency for each acquisition, providing 12 measures in total for each crossing of post-receptoral channel x stimulus frequency for each participant. For display of the fit of the model to the time-series data, and for voxel selection, we first regressed out the effect of the attention task, and then averaged across the six acquisitions for a given post-receptoral direction with stimulus ordering “A”, and the six acquisitions with stimulus ordering “B”, and concatenated these. The R^2^ value of the model fit to these average data (which excludes variance attributable to the attention task) was retained.

### Anatomical regions of interest

An anatomical model of the retinotopic organization of V1 cortex was used to define the location and eccentricity mapping of this cortical area for each participant (Benson et al., 2014). For the analysis across eccentricity, the V1 ROI was divided into six, log-spaced divisions of eccentricity (0, 2.8, 5.6, 11.25, 22.5, 45, and 90°). The LGN ROI was defined in CIFTI FSLR_32k space. We segmented the LGN using the Freesurfer thalamic segmentation algorithm (Iglesias et al., 2018). A warp was calculated between participant anatomy and the Montreal Neurological Institute (MNI) space, and the segmentation masks were moved to the MNI coordinates with nearest neighbor interpolation. The reason for this operation is because the CIFTI FSLR atlas uses MNI for the subcortical voxel maps. Within a given eccentricity band (or region mask), voxels with a time-series model fit of R^2^ > 0.1 were selected and averaged to obtain temporal sensitivity measurements.

### Modeling of the temporal sensitivity function

TSFs were fit with a difference-of-exponentials model (Hawken et al., 1996) developed from Watson’s temporal sensitivity model (Watson, 1986):

$$H_{center}\left(\omega \right)={\left(i2\pi \omega \tau_{center}+1\right)}^{-nc},$$

$$H_{surround}\left(\omega \right)=G_{surround}{\left(i2\pi \omega \tau_{surround}+1\right)}^{-ns},$$

$$H\left(\omega \right)=G\left(H_{center}-H_{surround}\right)$$

where nc and ns represent the order of the center (fast) and surround (slow) filters, fixed at 9 and 10, respectively (Watson, 1986). Variables that were free to vary included $\tau_{center}$, the multiplier of the $\tau_{surround}$ relative to the $\tau_{center}$, and the multiplier of the relative to the $H_{surround}$. Linear constraints were placed on the model parameters, and an additional non-linear constraint was added to ensure the model produced a unimodal fit. The amplitudes of BOLD fMRI response across temporal frequency were fit with this model using the Matlab *fmincon* routine. While the Watson model provided a good description of the temporal sensitivity functions in our data, we found the model to be over-parameterized, in that model fits did not provide unique parameter solutions. Consequently, the values of the parameters were not especially informative. We instead summarized the model fits by noting the amplitude and temporal frequency at which the maximum interpolated response was found.

### Statistical analysis

Goodness-of-fit for fMRI timeseries modeling was determined by R^2^. TSFs are shown with the median and interquartile range (IQR). Median and IQR amplitude and peak temporal frequency were determined by fitting 1,000 TSFs bootstrapped across acquisitions under each stimulus condition. A map of temporal sensitivity was also created. To do so, we fit the Watson temporal sensitivity function to the response data from each vertex, separately for each stimulus direction. At each vertex, we found the interpolated temporal frequency corresponding to the maximum response (expressed as log Hz). The set of frequency values across vertices was then z-transformed by subtracting the across-voxel mean and then dividing by the standard deviation. The three z-maps obtained from the three stimulus directions were then averaged to provide an overall map of relative peak temporal sensitivity. This analysis was conducted only for the posterior 1/3^rd^ of the cortical surface, as minimal responses were found anterior to this point.

### Model of retinal signals

We created a model of predicted retinal output to our stimuli, based upon previous *in vivo* measurements in the macaque from three classes of retinal ganglion cells (parasol, midget, small bistratified). These data were extracted from Figures 1a, b of Solomon and colleagues (2002) and Figures 9a, b and 13a of Solomon(2005). The electrophysiologic data were recorded in response to stimuli similar to those employed in our study (spatially uniform, 4.7° diameter modulations that targeted the DKL cardinal axes around a 2000 Troland background). Measurements were made for log-spaced temporal frequencies and contrasts from cells in three different retinal eccentricity bands (0–10°, 20–30°, >30°; only the first two eccentricity bands available for the bistratified responses). We averaged the “on” and “off” cell responses when both were provided. Amplitude was given in units of “gain”, defined as spikes per second per percent contrast of the stimulus relative to the maximum possible cone contrast available for a given stimulus modulation in cone contrast space (measured at the initial slope of the contrast response function).

To obtain the predicted response to our stimuli, we calculated the spikes / sec response that would be expected for the contrast level used for our stimuli, with the assumption that parasol cells saturate at 25% contrast (Lee et al., 1990). The response to the LMS modulation was taken as the sum of the midget and parasol achromatic responses. We assigned the RGC measurements to three eccentricity locations within the cell recoding bins (5°, 25°, and 40°). To produce the predicted retinal response integrated across cell populations, we scaled responses at each eccentricity by the total quantity of RGC receptive fields (Watson, 2014), the relative proportions of the different RGC classes (Dacey, 1993; Drasdo et al., 2007), and the change in annular retinal area with eccentricity. We characterized the model output by the amplitude and temporal frequency at the peak response.

### Code Accessibility

All software used for data processing and data analysis is publicly accessible. The software used for overall data analysis and figure generation is found here: https://github.com/gkaguirrelab/Patterson_2023_JNeurosci. The routine used for non-linear fitting of the BOLD fMRI data may be found here: https://github.com/gkaguirrelab/forwardModel. The model of retinal response may be found here: https://github.com/gkaguirrelab/rgcTemporalSensitivity.

## Results

We obtained BOLD fMRI data from two participants, P1 and P2, while they viewed a spatially uniform, wide field of flickering light. Our stimuli separately targeted the three post-receptoral channels (Figure 1a). The design of these stimuli accounted for variation in receptoral spectral sensitivity across retinal eccentricity (Figure 1b), and were designed to produce spatially uniform, high contrast on the targeted post-receptoral channel across the visual field (Figure 1c). Below we refer to these stimuli as being oriented in different “directions” along the cardinal axes of Derrington-Krauskopf-Lennie (DKL) space (Derrington et al., 1984). Across many fMRI acquisitions, these spectral modulations were flickered at log-spaced temporal frequencies ranging from 2 to 64 Hz (Figure 1d). An additional, “0 Hz” condition presented the steady stimulus background. Participants viewed the stimulus field within an MRI-compatible eye piece through a +36 or +32 D contact lens, increasing the effective stimulus radius to ~70° eccentricity.

### Variation in flicker frequency induces consistent variation in BOLD fMRI responses

**Figure 2.**
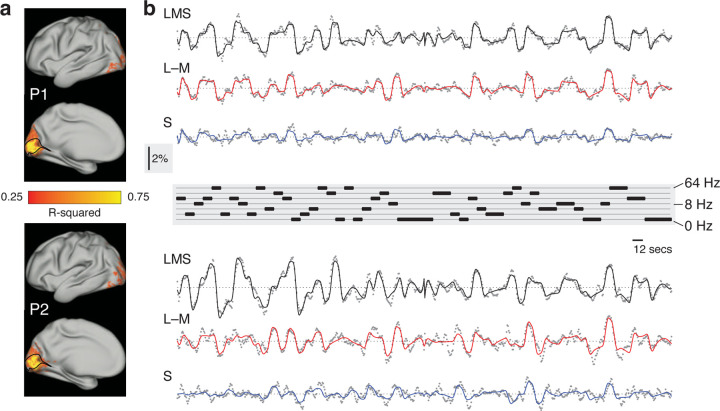
Fit to fMRI time series data The time series data at each voxel and vertex were analyzed for participant P1 (top) and P2 (bottom) across all acquisitions. (a) The partially inflated, pseudo-hemisphere, cortical surface map for each participant, with the R^2^ value of the model fit to the time series data at each vertex. A black line indicates the border of area V1, within which the highest model fit values were found. (b) The average BOLD fMRI time series (gray dots) across area V1, and across the acquisitions for each stimulus type (LMS, L–M, and S). The continuous line shows the fit of the time-series model. An inset, vertical scale bar at center-left indicates a 2% BOLD fMRI signal response. An inset scale bar at center-right indicates a 12 second interval. The plot at center provides the ordering of stimulus frequency. Inspection of the time series reveals both a systematic relationship with the stimulus sequence, and a similar form of response between the two participants.

First, we fit the BOLD fMRI data from each participant with a time-series model to recover the amplitude of response to each stimulus type at each voxel or vertex. The average amplitude of BOLD fMRI signal was estimated separately for each stimulus direction (LMS, L–M, S) for each stimulus frequency across the 12 second periods of stimulation; the amplitude of response to the attention events was also estimated. These estimates were made for each acquisition to allow assessment of measurement error. Figure 2a presents maps of the R^2^ fit of the time-series model for each participant across the cortical surface. A similar distribution of high-quality model fits was seen in both participants, largely confined to the early visual areas on the mesial cortical surface bilaterally. The highest model fit values were found within the borders of area V1.

We obtained the V1 time-series data and model fit (averaged across vertices and acquisitions) for each participant (after removing the effect of the attention events) for each of the three stimulus directions (Figure 2b). For both participants, the analysis provided a good description of the variation in the BOLD fMRI signal. Further, the time-series data were similar between the two participants, and systematically related to the pattern of stimulus temporal frequencies illustrated at center. There was clear variation in the amplitude of the cortical response as a function of the modulation frequency of the stimulus for each post-receptoral direction.

### Temporal filtering and amplification of chromatic signals between LGN and V1

**Figure 3.**
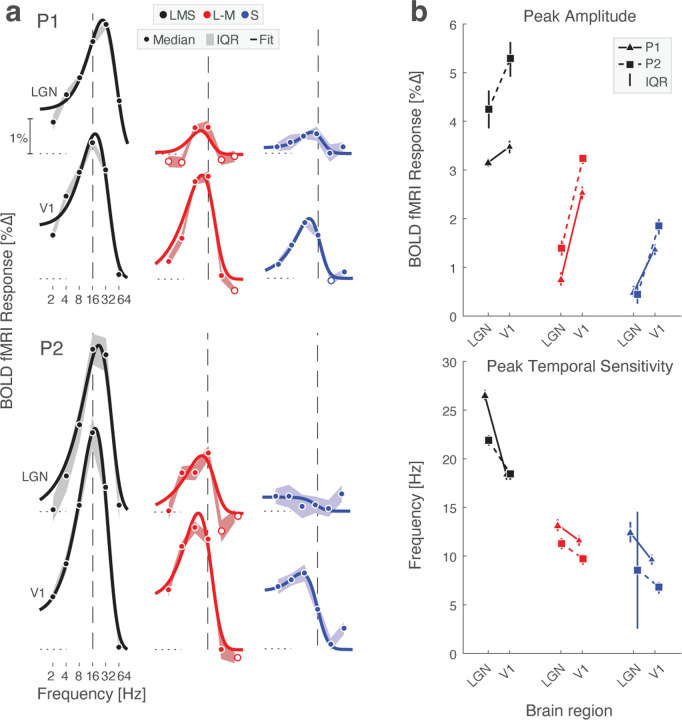
Temporal sensitivity to flicker in LGN and V1. (a) LGN and V1 TSFs for stimuli that target the 3 post-receptoral channels are shown for P1 (*top*) and P2 (*bottom*) fit with the Watson difference-of-exponentials model. Data points represent the boot-strapped median across acquisitions, and shading provides the interquartile range. A dashed reference line is positioned at 16 Hz in each plot, facilitating a comparison of the point of peak temporal sensitivity across plots. (b) Peak amplitude (top) and peak temporal sensitivity (bottom) for each subject across region. Peak amplitude and peak temporal sensitivity were determined by Watson model fits.

The first level analysis provided the amplitude of BOLD fMRI response to each stimulus frequency. We next created temporal sensitivity functions (TSFs) which describe the average response to each stimulation frequency (relative to the 0 Hz condition). Figure 3a presents the TSFs measured for each participant with the LGN and area V1. The data were fit with the Watson (1986) difference-of-exponentials temporal model, which provided a reasonable description. Visual inspection of the TSFs reveals two notable differences between LGN and V1 responses: (1) there was a relative increase in the amplitude of response to chromatic stimuli between LGN and V1; and (2) the peak temporal sensitivity shifted to lower frequencies between LGN and V1 across all post-receptoral channels. We quantified these impressions by obtaining the median (across bootstrapped acquisitions) peak amplitude and peak temporal sensitivity provided by the fit of the Watson model (Figure 3b). Larger responses were seen in V1 as compared to the LGN for all three post-receptoral channels, but this was more pronounced for chromatic stimuli, with an increase that was 2–3 times larger between these regions for chromatic as compared to achromatic stimuli. There was also a shift of peak temporal sensitivity towards lower frequencies between LGN and V1 for all three post-receptoral channels. This was largest for the achromatic stimuli, for which peak sensitivity dropped by 5–10 Hz between the thalamic and cortical sites.

### Peak temporal sensitivity in V1 slows towards the periphery

**Figure 4.**
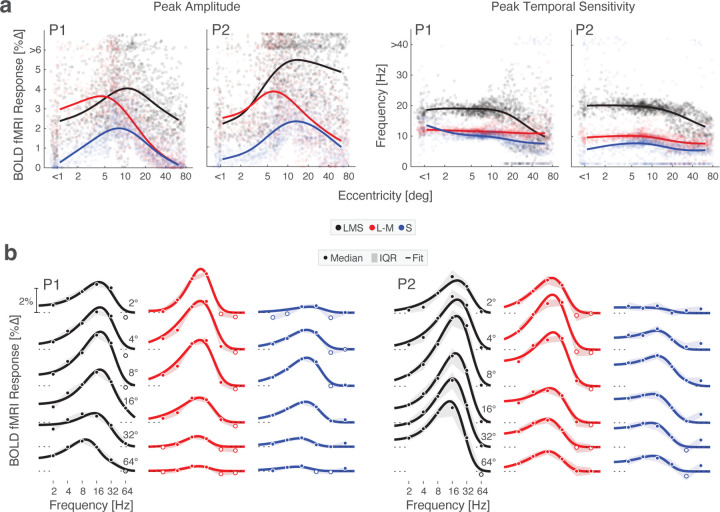
Temporal flicker sensitivity within area V1 across eccentricity. (a) Peak amplitude (right) and peak temporal frequency (left) within area V1 for P1 and P2 for stimuli that targeted each of the 3 post-receptoral directions. Dots represent values for each vertex and lines represent the fit of a smoothing spline to the median value at each of 30 eccentricity bins. (a) V1 TSFs at logarithmically spaced eccentricities across the 3 post-receptoral channels are shown for P1 (*left*) and P2 (*right*) with Watson temporal model fits. Data points represent the boot-strapped median across acquisitions, and shading provides the interquartile range.

The retinotopic organization of V1 allowed us to examine temporal sensitivity across eccentricity. BOLD fMRI responses at each vertex within V1 were fit with the Watson model to obtain the peak amplitude and peak temporal frequency of the TSF. We then examined how these properties varied across vertices as a function of eccentricity (Figure 4a). The interpretation of the absolute amplitude of response is complicated by the effect of non-neural influences upon the BOLD fMRI response; we nonetheless can examine the relative response between the stimulus directions. The response to the chromatic L–M modulation is comparable to the LMS-driven response out to ~5° eccentricity, and then drops steadily into the visual periphery. In contrast, the response to S-directed stimuli across eccentricity follows the same general form as the response to LMS, although is scaled down and reaches zero close to the fovea.

We examined peak temporal sensitivity across post-receptoral channel and eccentricity. Across eccentricity, higher peak temporal frequency values were generally found for the achromatic as compared to the chromatic stimuli. For the chromatic stimuli, there was only a small change in peak temporal frequency with eccentricity. In contrast, the peak response to LMS stimuli steadily shifted to lower frequencies starting at 10 – 20° eccentricity, eventually reaching the temporal sensitivity seen in response to L–M stimuli. Across the three post-receptoral directions, we found no evidence of a shift to higher temporal sensitivity with greater eccentricity.

Next, we divided area V1 into 6 concentric bands to visualize TSFs as a function of visual field eccentricity. To account for cortical magnification of the visual field, the borders of these bands were logarithmically spaced, yielding patches of cortex roughly matched in surface area. Within each band, we obtained the TSF for each stimulus modulation (Figure 4b). Generally, reliable TSFs were measured for both participants, for all three stimulus types, across eccentricity. This includes in the most peripheral eccentricity band (centered at 64°). Examination of these TSFs confirms the impression that the peak response to LMS stimuli shifts to lower temporal frequencies at greater eccentricities within area V1. This is evident both in the peak temporal frequency, as well as in a relatively larger low-pass component to the response.

### Peak temporal sensitivity increases beyond area V1

**Figure 5.**
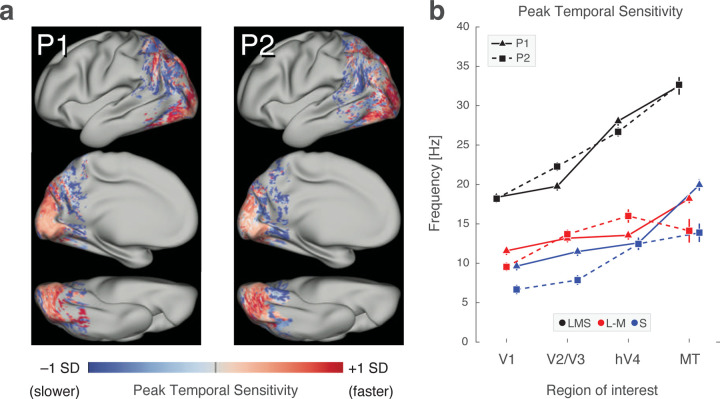
Peak temporal frequency beyond area V1. (a) Relative peak temporal sensitivity map across visual cortex, combined across stimulus directions for the two participants. Lateral, medial, and ventral views are provided. Map values reflect the relative peak frequency across the cortex within each of the stimulus directions. (b) Median temporal sensitivity (boot-strapped across acquisitions) for each of several visual cortex regions of interest. Error bars provide the interquartile range.

We created maps of peak temporal frequency across the cortex for each stimulus direction (Supplementary Figure 2). Although these maps differed in their absolute values, we found that the patterns of higher and lower temporal sensitivity were quite similar across the three stimulus directions. Therefore, we standardized the maps across vertices for each stimulus type and combined them (Figure 5a). A similar pattern of response was seen for both participants. Within area V1, there was a relatively uniform peak sensitivity to mid-range temporal frequencies. Within posterior extra-striate cortex, peak sensitivity shifted to higher temporal frequencies; this was the case for both dorsal and ventral visual areas. Finally, at more anterior points within visually responsive cortex, peak sensitivity was found at lower temporal frequencies.

We measured the peak temporal sensitivity of specific extra-striate regions (Figure 5b). For both participants across all three stimulus directions, peak temporal sensitivity increased across the visual hierarchy, rising between V1 and V2/V3, and again into both MT and hV4. For achromatic stimuli, MT demonstrated the highest peak temporal sensitivity; this pattern was not reliably seen in response to the chromatic stimuli.

### Comparison of V1 responses to predicted retinal signals

**Figure 6.**
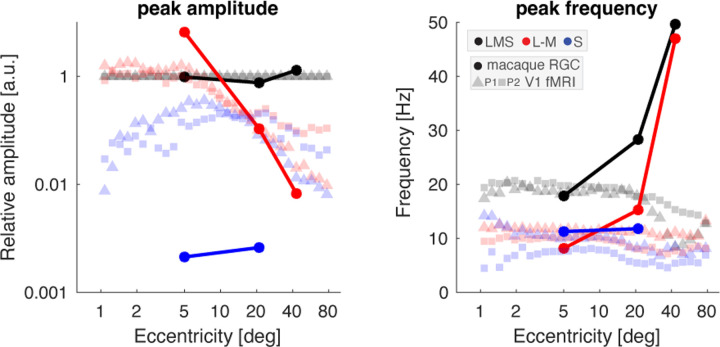
Predicted retinal signals compared to V1 response. Peak amplitude (left) and peak temporal frequency (right) of the V1 fMRI data from this study (squares and triangles) compared to predicted retinal output (filled circles) based upon physiologic recordings of macaque RGCs, integrated across the total cell population present at a given eccentricity location. Peak amplitude is expressed relative to responses to LMS-directed stimuli. For the retina model, normalization is to the average response evoked by LMS stimuli across eccentricity; for the V1 data, normalization is performed at each of 30 eccentricity bins.

To better understand the transformation of signals from the retina to the cortex, we generated a prediction of integrated retinal output to our stimuli, based upon prior measurements of macaque RGC responses (Solomon et al., 2002; Solomon, 2005). In Figure 6, the filled circles and lines correspond to the predicted retinal response, while the squares and triangles are the data measured from our two participants within area V1 across eccentricity.

The predicted amplitude of retinal response to LMS stimulation varies only a small amount among the three eccentricity locations for which measurements are available. This is in contrast to the response to L–M chromatic stimulation, which is initially greater than the response to LMS, but drops by an order of magnitude into the visual periphery, as the combined consequence of both a relative reduction in the midget RGC class and the reduced response per cell, perhaps due to the effects of random cone wiring (Wool et al., 2018). Finally, the predicted response to S-directed stimulation is markedly smaller due to the sparse population of small bistratified cells that are presumed to carry this signal.

We may compare these model values to the fMRI data, normalized at each eccentricity location by the response to LMS stimulation. Similar to the retinal prediction, the relative response to L–M stimulation drops steadily beyond 5–10° eccentricity, although the V1 response is smaller than might be predicted in central vision. The amplitude of response to S-directed stimuli within V1 is markedly larger than predicted by the retinal model, rising futher towards 5–10° eccentricity, then falling in concert with the response to L–M.

The retinal model predicts the temporal frequency that would elicit the peak response for each post-receptoral direction. At 5° eccentricity, there was rough agreement between the retinal model and the fMRI data. At greater eccentricities, however, retinal responses to LMS and L–M stimuli were predicted to shift to ever higher peak temporal frequencies, in marked contrast to the properties of the fMRI data.

## Discussion

Our study provides detailed temporal sensitivity measurements across subcortical and cortical regions in response to supra-threshold stimulation of the three canonical, post-receptoral visual channels. These results also characterize a fundamental aspect of human cortical visual function across eccentricity. Many of our findings replicate known properties of the visual system, including a relative amplification of chromatic signals between the LGN and area V1 (Cottaris and De Valois, 1998; Mullen et al., 2008), filtering of high temporal frequencies between these neural stages (Horwitz, 2021), attenuated responses to S-cone stimulation at the fovea (Williams et al., 1981), a systematic difference between chromatic and achromatic channels in peak temporal sensitivity (Swanson et al., 1987; Spitschan et al., 2016), and faster peak temporal sensitivity in extra-striate cortex (Spitschan et al., 2016). Contrary to what might be expected from the properties of the retina and threshold psychophysical measurements, however, we do not observe an increase in peak temporal sensitivity to achromatic stimuli in the visual periphery. Unexpectedly, we find a shift in these cortical responses to lower temporal frequencies.

### Comparison to prior neuro-imaging studies

There have been prior studies of the temporal sensitivity for achromatic and chromatic modulations within primary visual cortex (Engel et al., 1997; D’Souza et al., 2011) and extra-striate cortex (Spitschan et al., 2016), but these experiments did not examine responses across eccentricity. Chai and colleagues (2019) measured BOLD fMRI responses to a spatially structured, 15° radial eccentricity, achromatic checkerboard pattern that flickered between 1 to 40 Hz. Their parcellation of cortex by temporal sensitivity is similar in appearance to our temporal sensitivity map (Figure 5a). Kim and colleagues (Kim et al., 2023) developed a spatiotemporal receptive field model and analyzed fMRI data collected in response to drifting bars that contained multi-colored images. They find that a temporal integration parameter increases across the first 12 degrees of V1 eccentricity, although there is not a straightforward relationship between their model and our measurements.

Himmelberg and Wade (2019) reported that V1 response contrast sensitivity for a 20 Hz achromatic grating was greater at 6–10° eccentricity as compared to the fovea. As we did not measure a neural contrast response function for our stimuli, we are not able to directly compare this prior result to our own. Additionally, temporal sensitivity varies substantially with luminance and spatial frequency (Watson, 1986), and these stimulus properties differ between the current study and that of Himmelberg and Wade. We measured responses to a spatially uniform field at high retinal illuminance and took advantage of tailored spectral design to equate stimulus contrast across eccentricity. An advantage of this approach is that we do not need to contend with the effect of variation of acuity across the visual field (Virsu et al., 1982).

### Normalization of retinal signals in the visual cortex

Comparison of our measurements from area V1 to a predicted retinal response suggests that there is extensive post-retinal modification of visual signals across eccentricity. Variation in response amplitude in V1 across stimulus directions is a log unit less than predicted to arise in the retina. There is also a notable difference between predicted and observed temporal sensitivity with eccentricity: while the visual cortex data at 5° eccentricity is consistent with the retinal prediction, at further eccentricities cortical responses peak at far lower stimulation frequencies than predicted. It appears that post-retinal modification between the retina and V1 normalizes responses across eccentricity and post-receptoral channel, modulating the relative amplitude of chromatic responses and slowing peak temporal sensitivity towards the periphery. Overall, visual cortex responses substantially reduce the striking differences across post-receptoral channel and eccentricity observed in the retina.

We might consider alternative explanations for the relative stability of temporal frequency sensitivity that we observe across eccentricity in area V1. Our stimuli were wide-field and spatially uniform. We therefore derived temporal sensitivity measures from simultaneously stimulated eccentricities. It is possible that lateral or hierarchical influences between neurons across eccentricity locations cause homogenization of responses (Nassi et al., 2014). Alternatively, our stimulus might have been ineffective at driving neurons that have both narrower spatial tuning, and which vary in temporal tuning across eccentricity (Reinhard and Münch, 2021). In either case, measurements made with spatially restricted stimuli that produce isolated stimulation at different eccentricities might produce different findings.

We designed our stimulus to be spatially uniform, including accounting for variation in spectral sensitivity of the cone fundamentals with eccentricity (Supplementary Figure 1). Nonetheless, we may be concerned that stimulus contrast or background luminance declined into the periphery, causing a decrease in temporal sensitivity (Snowden et al., 1995). The absolute amplitude of BOLD fMRI response measured for all three stimulus directions decreased beyond ~10° eccentricity, potentially supporting this concern. We are reassured, however, that this effect is unlikely to account for the slowing of temporal sensitivity for the luminance channel, as the amplitude of response in the far periphery is equal or greater than found at the fovea, despite a slowing of the TSF. In future studies, measurement of temporal sensitivity at varying contrast levels could be used to better address this concern, and potentially disambiguate the effects of non-neural influences upon the BOLD fMRI response (Kurzawski et al., 2021).

### Relation to psychophysical measures

The cortical temporal sensitivity functions we have obtained here are in good agreement with known properties of human perception. This includes the both the general form of the responses—which are well-fit by the Watson difference-of-exponentials model—and the variation in peak temporal sensitivity between the chromatic and achromatic channels.

Perceptual temporal sensitivity across visual field eccentricity has been well studied for the detection of high-frequency achromatic flicker. Specifically, the critical flicker fusion (CFF) frequency is a measure of highest frequency stimulus (in the presence of noise) that can be distinguished from a static background. There is evidence that people are able to perceive higher frequency achromatic flicker in the periphery of their visual field (Hartmann et al., 1979; Tyler and Hamer, 1993), although it is unclear if this reflects a change in the shape of the entire sensitivity function or variation in the signal:noise ratio with eccentricity (Rider et al., 2021).

While we might consider measuring an analog of the CFF in our BOLD fMRI data, this analysis faces practical limitations. For almost all the measurements across eccentricity, the BOLD fMRI response to 32 Hz LMS flicker is far above threshold, while the response at 64 Hz is zero; the cortical CFF lies somewhere between these two frequencies. Therefore, we regard our data as not able address this question. Anecdotally, however, we note that one observer (P1) perceived a faint, shimmering flicker in an annular zone of their peripheral vision during presentation of the 64 Hz LMS stimulus. This participant was also found to have a non-zero cortical response to this same stimulus, only in the 16° eccentricity band. The second observer did not report this percept and did not have measurable BOLD fMRI responses to 64 Hz flicker within the V1 eccentricity bands.

Our study measured the supra-threshold cortical temporal sensitivity function. A study of supra-threshold temporal frequency discrimination using achromatic flicker found that performance at the fovea and at 30° eccentricity was quite similar (Waugh and Hess, 1994), perhaps consistent with our finding here that the supra-threshold temporal sensitivity of visual cortex changes minimally across eccentricity.

### Conclusions

This study provides the eccentricity dependence of the temporal sensitivity functions for the three cardinal post-receptoral channels within V1, and the spatially averaged sensitivity functions across multiple levels of the visual hierarchy. After the LGN, peak temporal sensitivity increases across the visual cortical hierarchy. We find that responses in primary visual cortex across post-receptoral channel and temporal frequency are normalized relative to predicted signals from the retina. This includes an unexpected slowing of cortical temporal sensitivity for achromatic flicker in the visual periphery.

## Supplementary Material

Supplement 1

## Significance Statement

We report the temporal sensitivity of human visual cortex across the three canonical post-receptoral channels from central vision to the far periphery. Functional MRI measurements of responses from the LGN, V1, and higher visual cortical areas demonstrate modification of temporal sensitivity across the visual hierarchy. This includes amplification of chromatic signals between the LGN and V1, and a steady increase in peak temporal sensitivity across visual areas beyond area V1. Within V1, we unexpectedly find that peak temporal sensitivity slows in the periphery, especially for achromatic stimuli. Comparison of our results to a model of retinal output demonstrates the substantial post-retinal filtering at work, yielding greater uniformity of responses across area V1 than would be predicted from unmodified retinal signals.

## Abbreviations:

LGN: Lateral geniculate nucleus
TSF: Temporal Sensitivity Function
RGC: Retinal ganglion cell
V1: Primary visual cortex
