# Supplementary material for: Temporal sensitivity for achromatic and chromatic flicker across the visual cortex
Source: bioRxiv. 2023 Jul 26:2023.07.24.550403. Preprint. [Version 1] doi: 10.1101/2023.07.24.550403 (PMC10402088; doi:10.1101/2023.07.24.550403)
Supplement: Supplementary file 1 [file media-1.pdf]

Temporal sensitivity for achromatic and chromatic flicker across the visual cortex

Supplementary Figures and Tables

Carlyn Patterson Gentile, Manuel Spitschan, Huseyin O. Taskin, Andrew S. Bock, Geoffrey K. Aguirre

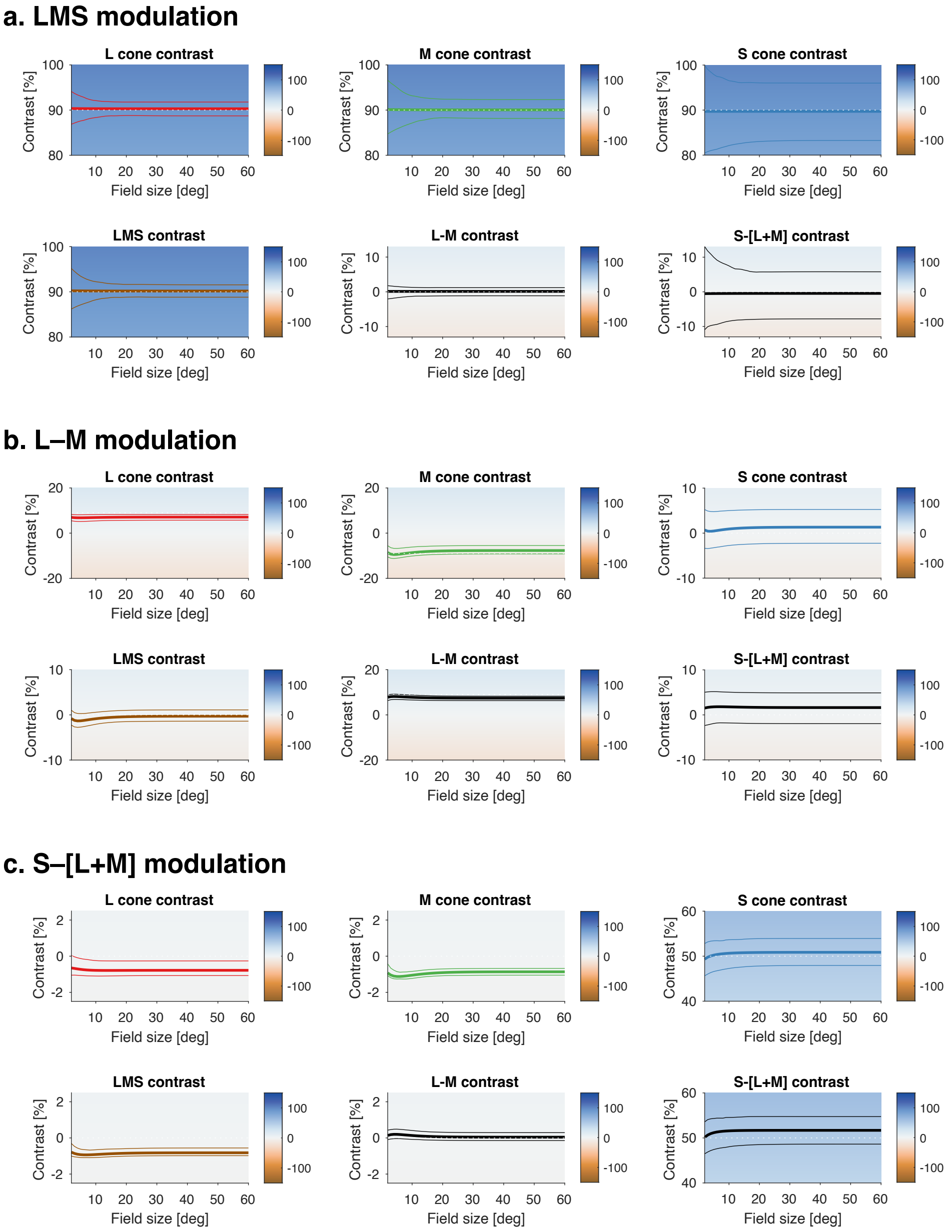

Supplementary Figure 1. Validated and inadvertent stimulus contrast
Spectroradiometric validation measurements of the stimuli were made before the first scanning session. The contrast produced by these stimuli upon the cones and their post-receptoral combinations was calculated for a nominal, 32 year old observer as a function of eccentricity (the thick line in each plot). The effect of biological variation in the assumed properties of the photoreceptors and pre-receptoral filtering was estimated by Monte-Carlo simulation, using previously described methods (Spitschan et al., 2017). The 95% confidence interval around the predicted contrast is given by the thin lines. Panels (a, b, c) correspond to the three stimulus modulations used in the experiment. The calculated contrast and confidence interval values at 2 and 30° is given in supplementary table below.

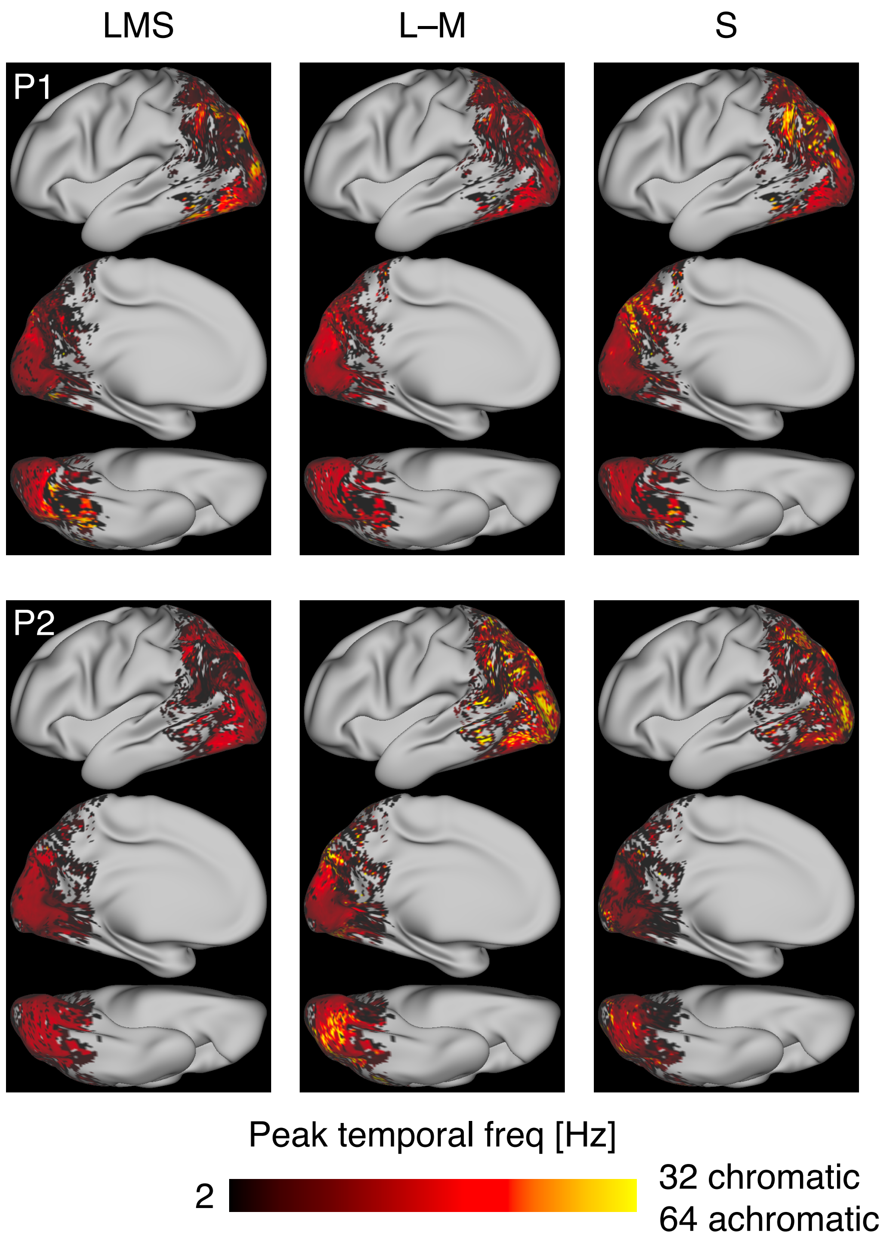

Supplementary Figure 2. Peak temporal frequency maps
The Watson difference-of-exponentials model was fit to the responses at each vertex for each of the three stimulus directions. Each map presents the temporal frequency at which the maximum amplitude of response was found. Responses from the left and right hemisphere were averaged into a pseudo-hemisphere. A different maximum frequency value is used for the color scale for the achromatic and chromatic maps.

Contrast at 2° eccentricity [95% CI]

**stimulus**	*LMS*	*L-M*	*S-[L+M]*
LMS	90.2 [86.2 95.2]	0.1 [-2.0 1.8]	-0.5 [-11.0 13.1]
L-M	-0.8 [-2.3 1.0]	7.8 [6.4 8.8]	1.5 [-2.4 5.0]
S-[L+M]	-0.8 [-1.0 -0.3]	0.1 [-0.1 0.4]	50.1 [46.5 53.6]

Contrast at 30° eccentricity [95% CI]

**stimulus**	*LMS*	*L-M*	*S-[L+M]*
LMS	90.2 [88.8 91.6]	0.1 [-1.1 1.2]	-0.5 [-7.8 5.8]
L-M	-0.3 [-1.4 1.1]	7.4 [6.3 8.3]	1.6 [-1.9 4.9]
S-[L+M]	-0.8 [-1.0 -0.6]	0.0 [-0.1 0.3]	51.7 [48.6 54.7]

Supplementary Table 1. Stimulus contrast at 2 and 30 degrees eccentricity

The values presented here correspond to the thick and thin lines from Supplementary Figure 1, at 2° and 30° on each plot that calculate contrast upon the post-receptoral channels. The cells that contain the contrast value for the targeted post-receptoral direction for each stimulus are indicated with red shading. Notably, there is minimal change in achieved contrast across a large range of eccentricity, and the off-diagonal contrast levels are uniformly low.

Reference

Spitschan M, Bock AS, Ryan J, Frazzetta G, Brainard DH, Aguirre GK. The human visual cortex response to melanopsin-directed stimulation is accompanied by a distinct perceptual experience. Proceedings of the National Academy of Sciences. 2017 Nov 14;114(46):12291-6.
